# Aggressive Systemic Mastocytosis in Association with Pure Red Cell Aplasia

**DOI:** 10.1155/2018/6928571

**Published:** 2018-07-08

**Authors:** Dhauna Karam, Sean Swiatkowski, Mamata Ravipati, Bharat Agrawal

**Affiliations:** ^1^Rosalind Franklin University, 3333 Green Bay Road, North Chicago, IL 60064, USA; ^2^Captain James A. Lovell Federal Health Care Center, 3001 Green Bay Road, North Chicago, IL 60064, USA

## Abstract

Aggressive systemic mastocytosis (ASM) is characterized by mast cell accumulation in systemic organs. Though ASM may be associated with other hematological disorders, the association with pure red cell aplasia (PRCA) is rare and has not been reported. Pure red cell aplasia (PRCA) is a syndrome, characterized by normochromic normocytic anemia, reticulocytopenia, and severe erythroid hypoplasia. The myeloid and megakaryocytic cell lines usually remain normal. Here, we report an unusual case of ASM, presenting in association with PRCA and the management challenges.

## 1. Introduction

Aggressive systemic mastocytosis is a rare disorder characterized by abnormal accumulation of mast cells in bone marrow and internal organs (liver, spleen, lymph nodes, and gastrointestinal tract) [1]. Mastocytosis was initially classified as one of the subtypes of “myeloproliferative neoplasms (MPN).” In the 2016 revision of the World Health Organization (WHO) classification of tumors of the hematopoietic and lymphoid tissues, mastocytosis was classified as a separate entity [2, 3]. Chemical mediators such as tumor necrosis factor produced by mast cells can suppress erythropoiesis, and some mast cell diseases can cause hypoplastic anemia, though the pathogenesis is not clear. Our case report highlights an unusual and rare presentation of ASM with PRCA. Such an association has not been reported in the literature, except in one case report where mast cell activation disorder and PRCA occurred together [4].

## 2. Case Presentation

### 2.1. Patient's Symptoms/History

A 64-year-old white male, with past medical history of depression, presented with progressive weakness, unintentional weight loss, and exercise intolerance since past 1 month. He was a very healthy and active person; he enjoyed biking and rollerblading. The above symptoms were very unusual for him. The patient reported intermittent episodes of epistaxis, 3-4 times a week since the past month, lasting for a few minutes. He also endorsed 2-3 episodes of loose stools daily since the past month. Of note, the patient had history of exposure to Agent Orange between years 1969 and 1971; the first exposure was forty-five years earlier. Physical examination revealed stable vital signs with a palpable spleen of six finger-breadths below the left costal margin and mild hepatomegaly. Cardiopulmonary, lymphatic, and dermatologic examination, including Darrier's sign were all negative.

### 2.2. Diagnosis

**Figure 1:**
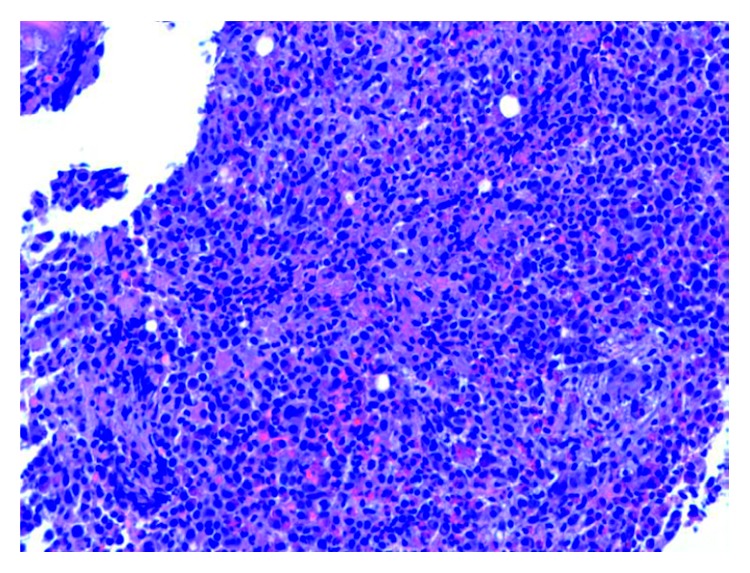
Area of hypercellular bone marrow with red cell aplasia.

**Figure 2:**
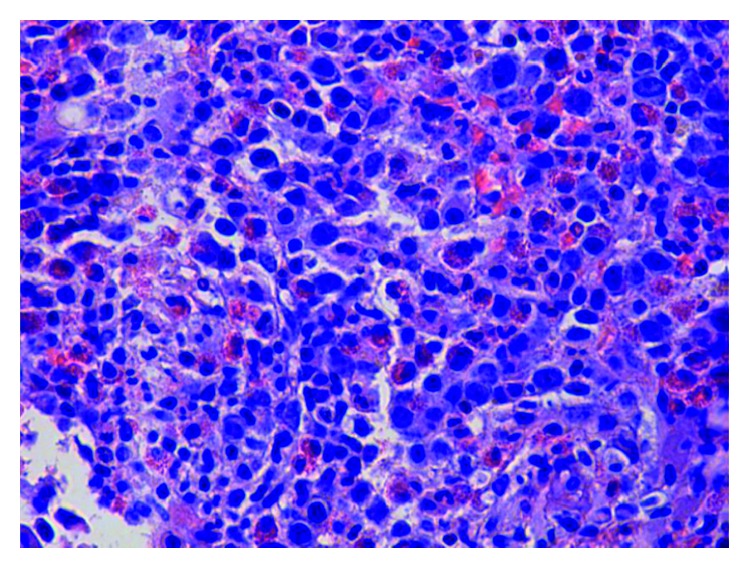
Bone marrow with hypercellularity and increased mast cells.

**Figure 3:**
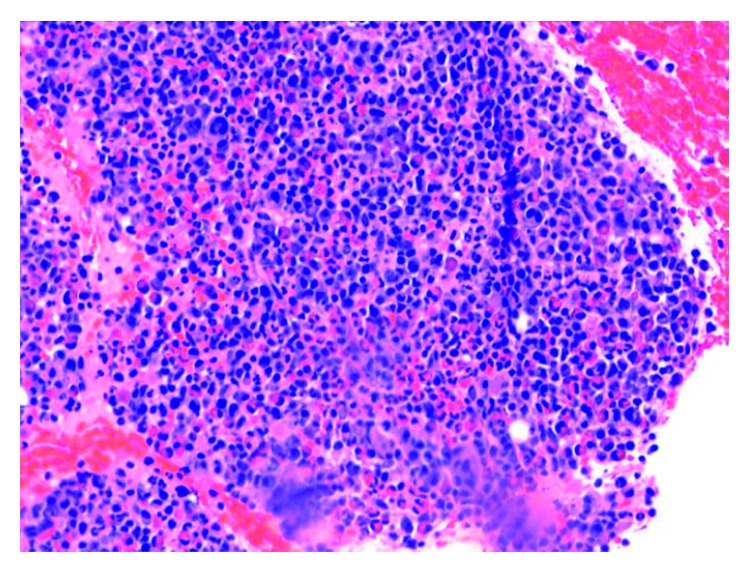
Clot section which shows hypercellularity and increased mast cells.

**Figure 4:**
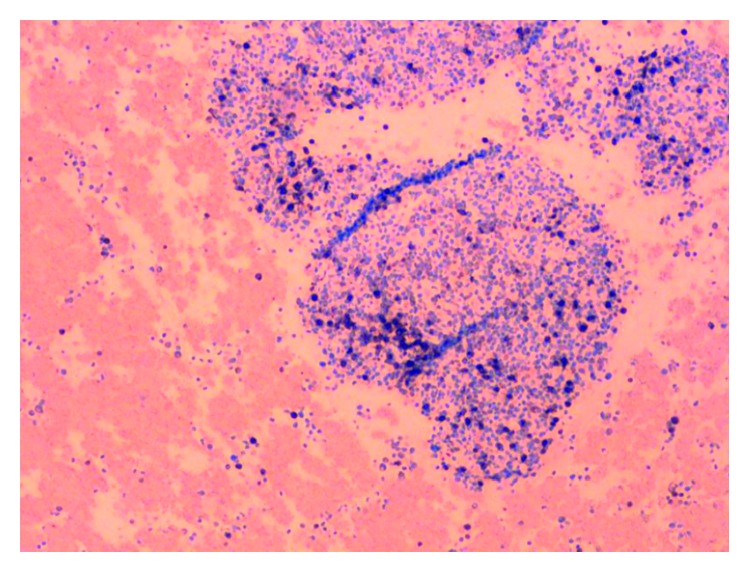
Clot stained with CD2 which is positive in mast cells.

**Figure 5:**
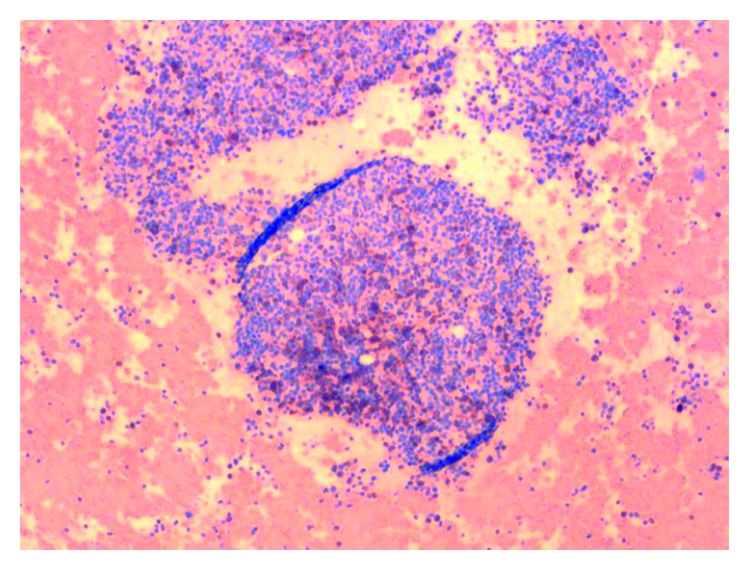
Clot stained with CD25 which is positive in mast cells.

On admission, the patient was found to have a hemoglobin count of 5.1 g/dl, which was a significant drop from the patient's baseline hemoglobin of 13-14 g/dl. Other basic laboratory studies are presented in Table 1. Urine analysis, stool for blood test, serum haptoglobulin, LDH, hepatitis B and C testing, PNH by flow cytometry, and hemochromatosis gene mutation were normal or negative. Serum tryptase level was elevated at 1110 ng/ml (normal < 11.4 ng/ml). Bone marrow biopsy and clot section performed as a part of anemia workup revealed hypercellularity with markedly increased maturing granulopoiesis with increased number of neutrophils and eosinophils. Erythropoiesis was markedly decreased with only very rare proerythroblasts present (Figure 1). Megakaryocytosis with dysmegakaryopoiesis was also appreciated. Several perivascular fibrotic areas containing mast cell aggregates were also identified (Figures 2 and 3). The mast cells were positive for mast cell tryptase and aberrant expression of CD2 and CD25 (Figures 4 and 5). c-KIT and D 816 V mutations were detected. The above findings were suggestive of ASM. FISH, cytogenetic, and flow cytometric analyses were unrevealing. Parvovirus immunostain was negative.

### 2.3. Treatment

After the diagnosis of ASM and PRCA, the patient left our hospital against medical advice. He became completely transfusion-dependent and was receiving weekly red cell transfusions from a nearby community hospital. He returned to our hospital after 5 weeks with worsening anemia, thrombocytopenia, liver function tests, and coagulopathy. The patient reported lack of transportation and social support to return to hospital for treatment. Peripheral smear revealed normochromic normocytic anemia with occasional ovalocyte, rare target cell, and dacrocyte. No nucleated red blood cell was identified. Granulocytes appeared mature without abnormal granulation or segmentation. Eosinophils were increased (30% with an absolute number of 1400/mcL) without abnormal features. Monocytes were increased to 12.9% without absolute monocytosis. Imaging studies (ultrasound and CT scan of abdomen) revealed hepatomegaly (liver 19.7 cm in length) and splenomegaly (spleen 19.1 cm in length) with multiple retroperitoneal lymph nodes.

With the patient's prior history of depression and ongoing thrombocytopenia, interferon alfa and 2-chlorodeoxyadenosine were not recommended for treatment of ASM. New avenues of treatments were discussed. Since the mast cells were CD30+, brentuximab vedotin was administered at a dose of 1.8 mg/kg. After therapy, patient developed worsening neutropenia, despite filgrastim and worsening anemia, and thrombocytopenia. The patient also developed Gram-negative bacteremia secondary to a urinary tract infection and became hypotensive and hypoxemic with lactic acidosis. The patient died 2 weeks later in the intensive care unit. Our patient lived for a short time after diagnosis of ASM; hence, there was not enough time for many sequential therapies. He did receive high-dose steroids before and concurrently with brentuximab therapy.

### 2.4. Outcome

The patient deceased within 2 months of initial diagnosis.

## 3. Discussion

Aggressive systemic mastocytosis is an uncommon disorder characterized by neoplastic mast cell accumulation in various organs. The skeletal system, bone marrow, gastrointestinal tract, and spleen are commonly involved. Mast cell infiltration of bone marrow leads to cytopenias, the so-called “C” findings [5, 6]. Systemic mastocytosis patients have poor prognosis because of multiorgan involvement and dysfunction [7].

Our patient presented with systemic symptoms and severe anemia, workup of which led to diagnosis of underlying systemic mastocytosis and pure red cell aplasia. The diagnosis of ASM was made by a constellation of clinical, cytogenetic, and molecular analyses [8]. Pure red cell aplasia was confirmed by normochromic anemia with very low reticulocyte percentage in presence of normal white cell and platelet counts, along with the finding of cellular marrow that revealed normal myelopoiesis, lymphopoiesis, and megakaryocytopoiesis, but very rare, if any erythroid precursors [9]. The association of mast cell disorder with pure red cell aplasia is rare and has been described only once in the literature [4].

Our patient had first exposure to Agent Orange 45 years earlier and then for the next three years. Agent Orange is a mixture of two chemicals that are phenoxy herbicides: 2,4 dichlorophenoxyacetic acid and 2,4,5 trichlorophenoxyacetic acid (2,4,5 T). The 2,4,5 T in Agent Orange was contaminated with small amount of dioxins. The main dioxin involved was 2,3,7,8-tetrachlorobenzo-p-dioxin or TCBD, which is one of the most toxic of dioxins and is classified as a human carcinogen by the U.S. Environmental Protection Agency. In the Vietnam War, between 1962 and 1971, the US military sprayed these herbicides. The Centre for Disease Control and Prevention notes that, in particular, there are increased number of cases of acute/chronic leukemias, Hodgkin's and non-Hodgkin's lymphomas, head and neck cancers, prostate cancer, lung/colon cancers, and soft tissue sarcomas occurring in the exposed population. Other reports have included multiple myeloma, AL amyloidosis, and other benign hematologic changes like anemia, leukopenia, and thrombocytopenia. Role of Agent Orange exposure in cytopenia or systemic mastocytosis in our patient remains of concern but cannot be stated with certainty. The complete blood count 2 years before diagnosis of ASM was normal in our patient.

The etiology of anemia in our patient was probably multifactorial: anemia of chronic disease/malignancy, bone marrow mastocytosis, splenomegaly, and pure red cell aplasia. Myelodysplastic syndrome (MDS) and autoimmune hemolytic anemia contributing to the patient's anemia could not completely be excluded. Serum protein electrophoresis was negative for monoclonal gammopathy, and quantitative measurements of IgG, IgA, and IgM were normal. Direct antiglobulin test was not performed initially. Despite frequent red cell transfusions, no crossmatching difficulties were reported by the blood bank. But, 3 weeks prior to the patient's demise, the blood bank reported difficulty in crossmatching for compatible red cells. A direct antiglobulin test performed then detected an IgG-negative complement-mediated positive test. The serum erythropoietin level was elevated at 1234 IU/L. High-dose methylprednisone 80–100 mg IV was initiated over the next week. In view of the severe reticulocytopenia and rising liver enzymes, coagulopathy as a result of hepatic involvement of ASM, response to steroids could not be determined. The patient remained transfusion-dependent and developed progressively severe pancytopenia.

Similarly, the etiology of thrombocytopenia was also multifactorial: infiltrating mast cells in the bone marrow and splenic sequestration. Increased megakaryopoiesis as well as dysmegakaryopoiesis in bone marrow raise the possibility of immune thrombocytopenia and MDS, respectively. MDS mutational analysis and FISH was performed to evaluate for critical regions in myelodysplastic syndrome which included deletion of 5q31 and 7q31, enumeration of chromosome 8, and deletion of long arm of chromosome 20. The studies were negative for all four regions, and JAK 2 study was also negative. Hence, in absence of abnormal cytogenetics, FISH, and flow cytometric studies, the diagnosis of MDS could not be confirmed. Flow cytometry did not demonstrate any increase in the blasts or immature cells. Immunostain for CD34 was positive only in rare cells in the bone marrow. FISH analysis for t(9 : 22) BCR/ABL 1 translocation, PDGFRA (4q12), FGFR 1(t 8 : 11), and PDGRB (5q33) rearrangement was negative. Mutational analysis for MDS detected mutation of ASXL1 and EZH2 genes. These genes are not specific for MDS and have been reported in many myeloid disorders as well as in ASM with unfavourable prognosis [10, 11]. Mutations in IDH1, IDH2, KRAS, NRAS, and TET 2 were negative.

The favoured diagnosis of bone marrow was ASM. The interpretation was supported by the presence of “C” findings including cytopenia indicating bone marrow dysfunction, in association with elevated liver enzymes suggesting liver damage and splenomegaly probably associated with hypersplenism. The patient also had diarrhea on presentation. As an infiltrative process, a proliferation of mast cells in the intestinal submucosa causes malabsorption. Release of histamine, both locally and systemically, other peptides, proteases, and generation of excessive quantities of mediators, such as prostaglandin D2, leukotriene C4, and platelet-activating factor are likely to alter gastrointestinal function and motility.

The patient presented with rapidly accumulating, extremely high iron saturation, and raised ferritin level, with negative mutation in HFE gene, which included C282 Y and H63D. Of note, iron saturation and ferritin levels were normal 4 months before diagnosis of ASM. Other causes of iron overload included ineffective erythropoiesis seen in MDS/sideroblastic anemia, thalassemia, and congenital or acquired hemolytic anemia associated with multiple transfusions. These conditions were unlikely in our patient because of the short time course for iron accumulation. A number of acute and chronic liver diseases causing liver inflammation can release stored iron into circulation raising serum ferritin [12]. One such inflammatory condition is hemophagocytosis syndrome (HPS). It is an extremely lethal condition in which excessive activation of immunity leads to tissue destruction. This condition was unlikely in our patient as the ferritin levels are usually over 5000 ng/ml in hemophagocytosis syndrome, whereas it was below 1000 ng/ml in our patient. The other feature of HPS patients is the acuity of illness with multiorgan involvement. Though our patient had multiorgan involvement from ASM, the cardinal features of HPS such as fever or rheumatologic symptoms were lacking, making the diagnosis unlikely in our patient. Malignancy can also be associated with elevated ferritin as suggested by a clinical trial published in 2015 [13]. Our patient had both an underlying malignancy and liver injury, the latter most likely contributing to the increased ferritin levels.

Management of any form of mastocytosis involves 3 different strategies: (a) general measures to prevent anaphylaxis, (b) antihistamine (cetirizine, hydroxyzine, and doxepin) and antileukotriene therapy (montelukast and zileuton) to treat symptoms associated with mast cell mediator release, and (c) cytoreductive therapy for advanced disease [10, 14, 15]. Midostaurin is a KIT inhibitor, first-line agent used in advanced disease, regardless of KIT mutation status [16]. Our patient did not receive the drug as it was approved by FDA only recently (April 2017). Tyrosine kinase inhibitors (TKI) such as imatinib have been used in ASM patients who do not have D816V mutation [17]. Our patient did express the D816V mutation and hence did not qualify for TKI therapy. At that time, the available cytoreductive therapies were interferon alfa and 2-CDA (chlorodeoxyadenosine). These drugs tend to have significant side effects with response lasting for short duration. This has led to an increasing need for novel agents with longer response and fewer side effects. Another potential therapeutic target CD30 (Ki-1) antigen was identified in patients with advanced ASM, and brentuximab vedotin has been used as an alternative therapy. Our patient received the same [18, 19]. Though brentuximab vedotin has a better safety profile, our patient was unable to tolerate even a single dose.

Cladribine is indicated in patients with rapidly progressive mastocytosis for rapid debulking and those who failed to respond to midostaurin or TKI. Hydroxyurea is also used in ASM patients, especially those with leukocytosis and/or splenomegaly-associated myeloproliferative neoplasms [20, 21]. None of the above therapies could be initiated in our patient due to worsening general condition after brentuximab. Hematopoietic stem cell transplant is the only curative option, though it is typically performed in younger adults [22]. PRCA, with or without ASM, is generally managed with regular transfusions for anemia, followed by immunosuppressive or cytotoxic therapy [23].

## Figures and Tables

**Table 1:** Laboratory values.

Laboratory analysis	Patient's values on initial hospitalization	Patient's values on second hospitalization	Normal range
Hemoglobin MCV MCHC	5.1 g/dl 97 fl 31.5 g/dl	7.4 g/dl 90.4 fl 32.9 g/dl	13–17 82–99 31–37
WBC count	6 k/*μ*L	5.1 k/*μ*L	4–10
Platelet count	91 k/*μ*L	90 k/*μ*L	150–400
Absolute eosinophil count	2 k/*μ*L	2 k/*μ*L	
Neutrophil	30%	30.8%	40–80
Lymphocyte	24%	23.5%	15–45
Eosinophil	33.5%	33.3%	0–6
Basophil	0%	0%	0–2
Glucose	140 mg/dl	117 mg/dl	70–99
BUN	16 mg/dl	14 mg/dl	7–21
Creatinine	0.87 mg/dl	0.83 mg/dl	0.67–1.17
AST	16 U/L	96 U/L	10–37
ALT	22 U/L	55 U/L	10–65
Alkaline phosphatase	475 U/L	130 U/L	50–136
Total bilirubin	0.8 mg/dl	21.3 mg/dl (direct 16.7 mg/dl)	0–1
Stool occult blood	Negative		
Haptoglobulin	214 mg/dl		30–200
Reticulocyte %	0.6		0.5–2.5
LDH	147 U/L		84–246
PT	12.8 s	27 s	9–12
INR	1.2		0.9–1.1
aPTT	33.1 s	77 s	23–34
Iron	202 *μ*g/dl		65–175
TIBC	203 *μ*g/dl		250–450
Iron saturation	100%		10–50
Ferritin	949.9 ng/ml		26–388
Vitamin B12	1680 pg/ml		193–986
Folate	19.6 ng/ml		8.7–55.4
TSH	1.48 uIU/ml		0.358–3.74
Fibrinogen		410	
SPEP—protein	6.8 g/dl		6.4–8.2
Albumin	2.8 g/dl	1.3 g/dl	3.5–5.0
Alpha-1 globulin	0.5 g/dl		0.2–0.4
Alpha-2 globulin	1.0 g/dl		0.5–1.0
Beta globulin	0.7 g/dl		0.5–1.1
Gamma globulin	1.8 g/dl		0.6–1.5

## Data Availability

The published data used to support the findings of this study (case report) are included within the article.
